# S100A14 Interacts with S100A16 and Regulates Its Expression in Human Cancer Cells

**DOI:** 10.1371/journal.pone.0076058

**Published:** 2013-09-27

**Authors:** Dipak Sapkota, Daniela Elena Costea, Salah O. Ibrahim, Anne C. Johannessen, Ove Bruland

**Affiliations:** 1 Department of Clinical Medicine, the Gade Laboratory for Pathology, University of Bergen, Haukeland University Hospital, Bergen, Norway; 2 Department of Clinical Dentistry, University of Bergen, Bergen, Norway; 3 Department of Pathology, Haukeland University Hospital, Bergen, Norway; 4 Center of Medical Genetics and Molecular Medicine, Haukeland University Hospital, Bergen, Norway; Chinese Academy of Medical Sciences, China

## Abstract

Both S100A14 and S100A16 are members of the multifunctional S100 protein family. Formation of homo/heterodimers is considered to be one of the major mechanisms for S100 proteins to execute their diverse cellular functions. By employing a classical Yeast two hybrid (Y-2 H) screen, we identified S100A16 as the single interaction partner of S100A14. This interaction was verified by co-immunoprecipitation, double indirect immunofluorescence and double immunostaining in specimens of oral squamous cell carcinoma and normal oral mucosa. The functional significance of this interaction was examined by employing retroviral mediated over-expression and knock-down of these proteins in several cancer cell-lines. Over-expression and knock-down of S100A14 led to concomitant up- and down-regulation of S100A16 protein in the cell-lines examined. However, there was no up-regulation of S100A16 mRNA upon S100A14 over-expression, indicating that modulation of S100A16 expression was not due to enhanced transcriptional activity but possibly by post-transcriptional regulation. In contrary, over-expression of S100A16 was associated neither with the up-regulation of S100A14 mRNA nor its protein, suggesting a unidirectional regulation between S100A14 and S100A16. Cellular treatment with protein synthesis inhibitor cycloheximide demonstrated a time-dependent intracellular degradation of both S100A16 and S100A14 proteins. Additionally, regulation of S100A16 and S100A14 degradation was found to be independent of the classical proteasomal and lysosomal pathways of protein degradation. Further studies will therefore be necessary to understand the functional significance of this interaction and the mechanisms on how S100A14 is involved in the regulation of S100A16 expression.

## Introduction

The S100 protein family is a multifunctional group of EF-hand calcium binding proteins. This family consists of small acidic proteins (10-12 kDa) that are expressed only in vertebrates in a cell and tissue specific manner. To date, 25 S100 protein members have been described in humans. Members of the S100 protein have been shown to regulate a number of biological processes like cell cycle, cell motility, signal transduction, protein phosphorylation, transcription, cell survival and apoptosis, related to normal development and carcinogenesis [1,2]. Despite these diverse functional roles, S100 proteins do not possess any enzymatic activities to account for their cellular activities [3,4]. One of the mechanisms for their varied cellular functions is the ability of the majority of S100 proteins to interact directly with a number of other cellular proteins, thereby modulating their functions [3]. Several members of S100 proteins can form homodimers/oligomers (for example: S100A4 [5], S100B [6]) or heterodimers (for example: interactions between S100A8 and S100A9 [7], between S100A4 and S100A1 [8], between S100B and S100A6 [6]) and these homo/heterodimer- formation is considered to be important for their cellular functions.

S100A14 is a recent addition to the S100 protein family [9,10]. Differential expression of S100A14 has been reported in a number of human cancers [9,11]. We have recently reported a role of S100A14 in the regulation of proliferation and invasion of oral squamous cell carcinomas (OSCCs) [12,13]. S100A14 was found to modulate expression of several molecules including p21, MMP1 and MMP9 in OSCC-derived cells [12,13]. In addition, the biological role of this protein has been reported in other human cancers, such as esophagus [14] and colon cancers [15]. However, the precise mechanism and molecular signaling of S100A14 in human cancer is not fully understood. Being predominantly a membranous protein [12] with N-myristoylation site at the N-terminus [9], it can be speculated that S100A14 might interact with other proteins potentially involved in signal transduction. Accordingly, S100A14 has been shown to interact in a calcium dependent manner with nucleobindin [10], a protein suggested to be involved in G protein-coupled signal transduction [16]. However, the ability of S100A14 to form heterodimers with other S100 proteins has not been examined. In this study, we show that S100A14 interacts with another S100 protein, the S100A16, and modulates its expression in human cancer cell lines.

## Materials and Methods

### Cell culture and treatments

The oral squamous cell carcinoma-derived cell-lines : CaLH3 [17], H357 [18], VB6 [19] and OSCC1 [13] were cultured as described previously [12]. HeLa cells (ATCC, CCL-2^TM^) were routinely maintained in Dulbecco’s modified Eagle’s medium (cat no: D6429, Sigma) supplemented with 10% FCS. All cell-lines were grown in humidified environment with 5% CO_2_ at 37 °C. Twenty four hours before the treatment with inhibitors, 1.0x10^5^ to 1.5x10^5^ cells per well were plated in 6-well plates. Cells were treated with 0, 50, 100 or 150 µM proteosomal inhibitor MG-132 (cat no: C2211, Sigma) for 5 and 10 hours; 0, 50, 100 or 150 µM lysosomal inhibitor chloroquin (cat no: C6628, Sigma) for 24 hours and 200 µg/mL protein synthesis inhibitor cycloheximide (cat no: #239763, Calbiochem) for 0, 3, 5, 7 and 8 hours. Following treatments, cells were lysed with 1X RIPA buffer and used for immunoblotting. Expression of S100A16 protein was calculated relative to the expression of GAPDH levels (using Multi Gauge software, Version 3.2, Fujifilm Corporation) and graphed as percent S100A16 protein remaining after cycloheximide treatment (data not shown). S100A16 half-life (*t*1/2) was calculated using the GraphPad prism 5.03 for Windows (GraphPad Software, San Diego California USA, www.graphpad.com).

### Yeast two-hybrid screen (Y-2H screen)

The coding sequences of human S100A14 were fused in frame with the GAL4 DNA-binding domain of the pGBT9 vector to generate pGBT9-S100A14 bait construct. Y-2H screening service was purchased from German Cancer Research Center, Heidelberg, Germany. Briefly, PGBT9-S100A14 construct was co-transfected with human cDNA keratinocyte libraries in *Saccharomyces cerevisiae*. Positive clones were identified by TRP1 selection at 0 mM 3-aminotriazol. All positive clones were sequenced by Sanger sequencing. The details of the pGBT9-S100A14 bait construction and Y-2H screening protocols are available upon request.

### Co-immunoprecipitation (Co-IP)

CaLH3 cells were lysed in ice cold lysis buffer (1X RIPA buffer, cat no: 89901, Pierce Biotechnology), supplemented with protease (Halt protease inhibitor cocktail kit, cat no: 78410, Pierce Biotechnology) and phosphatase (Phosphatase inhibitor cocktail 2, cat no p 5726, Sigma) inhibitors. Cell extracts were precleared by incubating with Protein A/G PLUS-Agarose (cat no: sc-2003, Santa Cruz) for 30 minutes. Five hundred micrograms of precleared cell extract was incubated with 3 µg of polyclonal rabbit anti human-S100A14 (cat no: 10489-1-AP, Proteintech) or polyclonal rabbit anti human-S100A16 antibody (11456-1-AP, Proteintech) for 1 hour at 4°C on a rotating wheel in the presence of 0-2 mM CaCl_2_ or 2 mM CaCl_2_ with 5 mM EDTA. Only lysate (without any antibody) and polyclonal anti-MMP9 antibody (cat no: sc-10737, Santa Cruz; isotype matched IgG) were used as controls for the Co-IP. Thereafter, 70 µL of resuspended Protein A/G PLUS-Agarose (cat no: sc-2003, Santa Cruz) was added to the lysate-antibody mix and incubated overnight at 4°C on a rotating wheel. After incubation, the beads were centrifuged at 1000xg for 5 minutes at 4°C and the supernatant was discarded. Beads were washed 4 times with PBS, resuspended and boiled at 90 4°C for 5 min in 30 µL SDS sample buffer and immunoblotted with polyclonal rabbit anti human-S100A16 (11456-1-AP, Proteintech, Chicago, IL, USA, 1:500 dilutions) or polyclonal rabbit anti human-S100A14 antibody (10489-1-AP, Proteintech, 1:1000 dilutions).

### Construction and transfection of expression and shRNA vectors

S100A14 expression vector was constructed as described previously [12]. Human cDNA encoding S100A16 was amplified using primer pairs (F: 5' –ATCCCGCGGCAGGGAGATGTCAGACTGCTA-3' and R: 5'-TGAGGATCCCTAGCTGCTGCTCTGCTG-3') and subcloned into the pRetroX-IRES-ZsGreen1 retroviral expression vector (Clontech Laboratories, Inc., CA, USA). shRNA targeting *S100A14* mRNA was constructed using the following oligonucleotides: shRNA1(F: 5’- GATCCCCTTGTGTAAGAGCCAAAGAATTCAAGAGATTCTTTGGCTCTTACACAATTTTTGGAAA-3’; R: 5’- AATTTTTCCAAAAATTGTGTAAGAGCCAAAGAATCTCTTGAATTCTTTGGCTCTTACACAAGGG-3’), shRNA2 (F: 5’- GATCCCCAGGGTCTTTAAGAACCTACTTCAAGAGA GTAGGTTCTTAAAGACCCTTTTTTGGAAA-3’; R: 5’- AATTTTTCCAAAAAAGGGTCTTTAAGAACCTAC TCTCTTGAAGTAGGTTCTTAAAGACCCTGGG-3’), shRNA3 (F: 5’- GATCCCCGAACCTACTTCCTAATCTCTTCAAGAGAGAGATTAGGAAGTAGGTTCTTTTTGGAAA-3’; R: 5’- AATTTTTCCAAAAA GAACCTACTTCCTAATCTCTCTCTTGAAGAGATTAGGAAGTAGGTTCGGG-3’). Oligonucleotides were annealed and inserted in the RNAi-Ready pSIREN-RetroQ-DsRed-Express expression vector (cat. no: 632487, Clonetech). shRNA targeting *LacZ* gene was used as a control for the S100A14-shRNAs. Cancer cell-lines were infected with the retroviruses derived from packaging (Phoenix A) cells, sorted (GFP/DsRed as a marker), propagated, verified for over-expression and knockdown of the respective proteins and used for functional assays.

### RNA extraction, cDNA synthesis and quantitative RT-PCT (qRT-PCR)

Total RNA was extracted from the cancer cell-lines using RNeasy fibrous tissue mini kit protocol (cat no: 74704, Qiagen Inc., Valencia, CA, USA). Following manufacturers’ instructions, 600 nanograms of total RNA was converted to cDNA using High-Capacity cDNA Archive Kit system (cat no: 4368814, Applied Biosystems, Foster City, CA, USA). All qRT-PCR amplifications were performed on ABI Prism Sequence Detector 7900 HT (Applied Biosystems, Foster City, USA) as described previously [12]. TaqMan assay for *S100A16* (Hs00293488_m1) and SYBR green based qRT-PCR (using the same primer pairs as for the construction of S100A14 expression vector) was performed to quantify *S100A16* and *S100A14* mRNAs in the cancer cell-lines. Comparative 2^-ΔΔ Ct^ method was used to quantify the relative mRNA expression.

### Immunoblot

Twenty five to forty µg of protein was resolved in NuPAGE® Novex 4-12% or 10% Bis-TrisTris gel (Life Technologies, NY, USA) in NuPAGE® MOPS/MES SDS running buffer (Life Technologies, NY, USA) and immunoblotted with polyclonal rabbit anti human-S100A14 (10489-1-AP, Proteintech, Chicago, IL, USA, 1:1000 dilutions), polyclonal rabbit anti human-S100A16 (11456-1-AP, Proteintech, Chicago, IL, USA, 1:500 dilutions) and monoclonal mouse anti human-p53 (sc-263, Santa Cruz Biotechnologies, CA, USA, 1:1000 dilutions) following standard western blot protocol. Anti-GAPDH (sc-25778, Santa Cruz, 1:5000 dilutions) was used as a loading control. Blots were visualized using enhanced chemiluminescence (Supersignal^®^ West Pico; Pierce Biotechnology, Rockford, IL, USA) and images were detected on a Fujifilm Las-4000 scanner.

### Double indirect immunofluorescence (DIF)

Use of OSCCs and their corresponding normal mucosa for DIF and immunohistochemistry was approved by the Regional Committee for Medical and Health Research Ethics, Western-Norway. Written consent was obtained from the participant of the study. Five µm sections of OSCCs and their corresponding normal mucosa were de-paraffinized, rehydrated following standard procedures and heat-induced epitope retrieval was performed in Tris-EDTA buffer (pH 9.0) by using microwaves (cat no: NN-L564W, Panasonic, Osaka, Japan). Afterwards, first primary antibody (polyclonal rabbit anti- S100A16 antibody, 11456-1-AP, Proteintech, 1:50 dilutions) was applied for 1 hour at room temperature and washed. The specimens were incubated with Fab fragment Goat anti-Rabbit IgG (cat no 111-007-003, Jackson ImmunoResearch, 1:50 dilutions) for 2 hours, to allow for switching of the rabbit IgG to goat Fab [20]. Thereafter, specimens were incubated with Mouse anti-GoatDyLight 488IgG (cat no 205-485-108, Jackson ImmunoResearch, 1:50 dilutions) secondary antibody for 1 hour, washed, blocked with 10% goat serum for 1 hour and the second primary antibody, polyclonal rabbit anti-S100A14 (cat no: 10489-1-AP, Proteintech, 1:600 dilutions), was applied for 1 hour at room temperature. After washing, secondary antibody Goat anti-Rabbit Alexa fluor^®^ 568 (cat no: A-11011, Invitrogen, 1:200 dilutions) was applied for 1 hour, washed and the slides were mounted in ProLong^®^ Gold antifade reagent with DAPI (cat no: P36935, Invitrogen). Specimens were examined using Leica TCS SP2 AOBS (Leica Microsystems, Germany) confocal laser microscope.

### Single and double immunohistochemistry (IHC)

Single and double IHC were performed on the serial sections of OSCCs and their corresponding normal mucosa. Single IHC for S100A16 and S100A14 was performed as described previously [12]. For double IHC, five µm sections were de-paraffinized, rehydrated following standard procedures and heat-induced epitope retrieval was performed in Tris-EDTA buffer (pH 9.0) by using microwaves (cat no: NN-L564W, Panasonic, Osaka, Japan). After blocking with peroxidase block and 10% goat serum, sections were incubated with polyclonal rabbit anti-S100A16 (cat no: 11456-1-AP, Proteintech, dilutions 1:200) for 1 hour at room temperature, washed and anti-rabbit secondary antibody conjugated with horseradish peroxidase labeled polymer (cat no: K4011, DAKO, Golstrup, DK) was applied and the reaction was visualized using ImmPACT VIP (cat no: SK-4605, Vectorlabs, California, USA) as enzyme substrate, resulting in a purple reaction product. To prevent cross reactivity with subsequent immunohistochemical reaction, antibodies of the first reactions were denatured by heating the specimens in pH 6.0 Target Retrieval Buffer (cat no: S1699, DAKO, Golstrup, DK) using microwaves (1000 Watts for 2 minutes and 250 watts for five and half minutes; NN-L564W, Panasonic, Osaka, Japan) [21]. Afterwards, sections were incubated with peroxidase block and 10% goat serum once again and the second primary antibody (polyclonal rabbit anti-S100A14, 10489-1-AP, Proteintech, dilutions 1:1400) was applied and washed. Thereafter, anti-rabbit secondary antibody conjugated with horseradish peroxidase labeled polymer (cat no: K4011, DAKO, Golstrup, DK) was applied and visualized with ImmPACT SG (cat no: SK-4705, Vectorlabs, California, USA) resulting in a gray reaction product. Specimens were then counterstained with hematoxylin (cat no: S3301, DAKO, Golstrup, DK), dehydrated and mounted with EuKit mounting medium. Specimens were examined using Leica DMLB microscope.

### Statistics

Data are expressed as mean ± standard error of the mean (SEM). For statistical analyses, Student’s-*t* test (paired) was performed using GraphPad prism 5.03 for Windows (GraphPad Software, San Diego California USA, www.graphpad.com), with the level of significance set at 5%.

## Results

### S100A16 was identified as an interaction partner of S100A14 both *in vivo* in the yeast cells and *in vitro* in the oral cancer-derived cells

**Figure 1:**
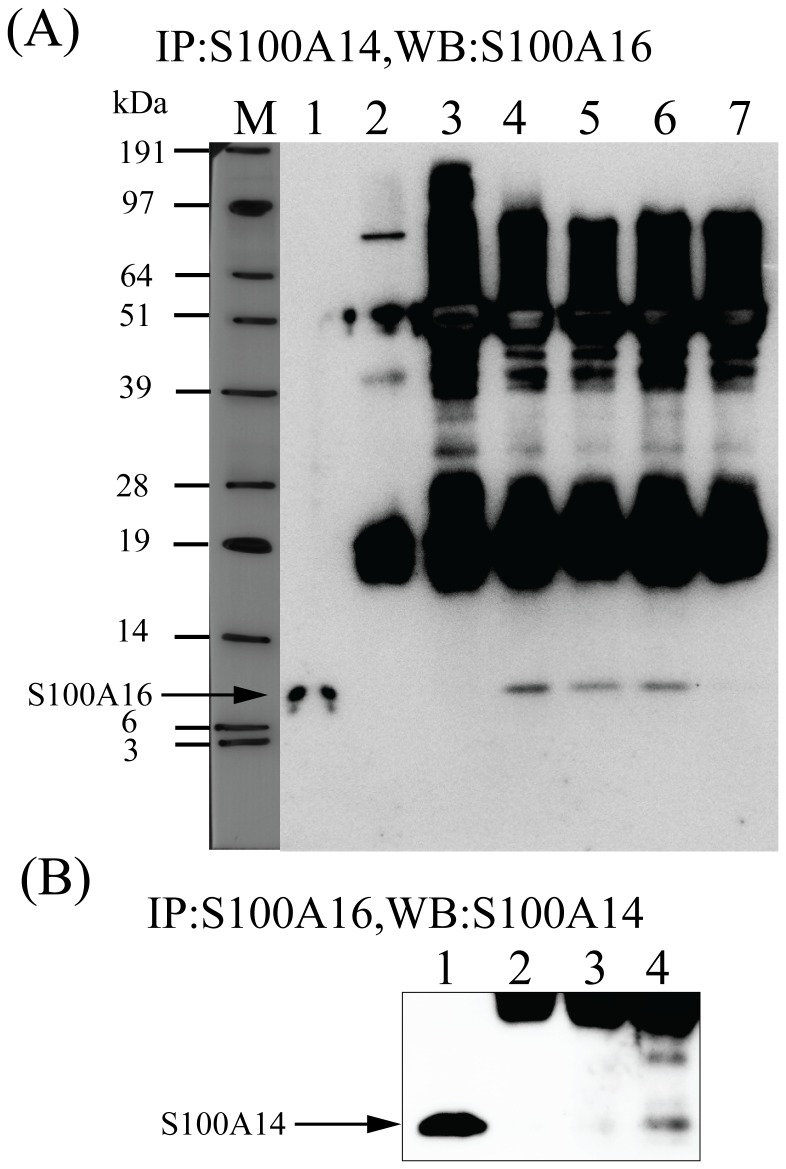
Co-immunoprecipitation of S100A14 and S100A16 in oral cancer cells. Five hundred micrograms of pre-cleared cell extract from CaLH3 cells was incubated with anti-S100A14 antibody in the presence of 0-2 mM CaCl_2_ or 2 mM CaCl_2_ with 5 mM EDTA (A) or anti-S100A16 antibody (B), followed by incubation with Protein A/G PLUS-Agarose. Immune complexes were immunoblotted with anti-S100A16 (A) or anti-S100A14 antibody (B). Only lysate (without any antibody) and anti-MMP9 antibody were used as controls for the Co-IP. (A) M, molecular weight marker; lane 1, input; lane 2, only lysate; lane 3, control anti-MMP9 antibody; lanes 4-7 (anti-S100A14 antibody): lane 4, 0 mM CaCl_2_; lane 5, 0.5 mM CaCl_2_; lane 6, 2 mM CaCl_2_; lane 7, 2 mM CaCl_2_ with 5 mM EDTA. (B) lane 1, input; lane 2, only lysate; lane 3, control anti-MMP9 antibody; lane 4, anti-S100A16 antibody with 0 mM CaCl_2_.

To identify interacting partners of S100A14, a human cDNA keratinocyte library was screened with the pGBT9-S100A14 bait construct in *Saccharomyces cerevisiae*. Out of 54 positive clones sequenced, human S100A16 was identified 53 times. DCAF8 was identified to be a false positive interaction partner of S100A14. Interaction between S100A14 and S100A16 proteins was verified by co-immunoprecipitation using the oral cancer derived CaLH3 cell-line (Figure 1A). Although previous reports showed enhanced interaction between other S100 proteins in presence of increasing Ca^2+^ concentrations, we could not detect increased immunoprecipitation between S100A14 and S100A16 with increasing Ca^2+^ concentrations (0.5 and 2 mM of CaCl_2_) (Figure 1A). However, no interaction was observed between S100A14 and S100A16 when 5mM of divalent metal ion chelator EDTA was used along with 2 mM of CaCl_2_ (Figure 1A). Reverse co-immunoprecipitation assay using specific antibody for S100A16 further confirmed the interaction between S100A14 and S100A16 proteins (Figure 1B).

### S100A14 co-localizes with S100A16 in specimens of OSCCs and normal oral mucosa

**Figure 2:**
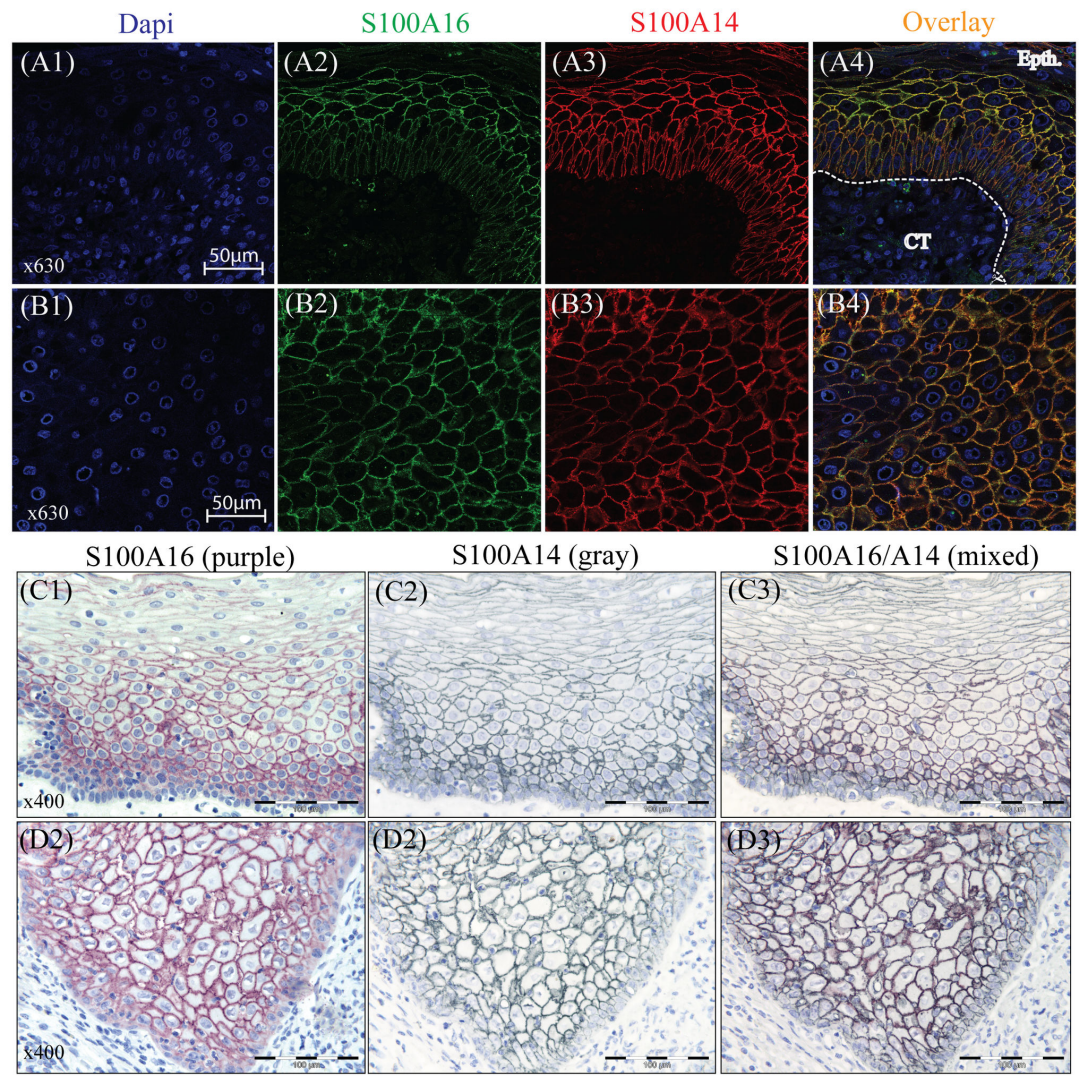
Co-localization of S100A14 and S100A16 in the specimens of OSCC and normal oral mucosa. Cellular localization of S100A14 and S100A16 was examined by performing DIF and IHC in the sections of formalin fixed and paraffin embedded OSCC and normal oral mucosal tissues. Predominantly membranous localization of S100A16 (green), S100A14 (red) and S100A16/A14 (yellow) was visualized in the epithelial cells of normal mucosa (A1-A4) and OSCC (B1-B4) tissues by DIF (Epth, epithelium; CT, connective tissue). Similarly, with single and double IHC on the serial sections, predominantly membranous expression of S100A16 (purple), S100A14 (gray) and S100A16/A14 (mixed color) was detected in the normal mucosa (C1-C3) and in the OSCC (D1-D3).

DIF and double IHC were performed in OSCCs and their corresponding normal mucosa to examine the sub-cellular localization of S100A14 and S100A16. DIF showed membranous co-localization of S100A16 and S100A14 proteins in the epithelial cells of both normal oral mucosa (Figure 2A1-A4) and OSCC (Figure 2B1-B4). Parallel to these results, membranous co-localization of these proteins was also observed on the serial sections of normal oral mucosa (Figure 2C1-C3) and in the invading island of OSCC (well differentiated lesion) (Figure 2D1-D3) by single and double IHC.

### S100A16 protein but not mRNA expression is regulated by S100A14 in different human cancer cell-lines

**Figure 3:**
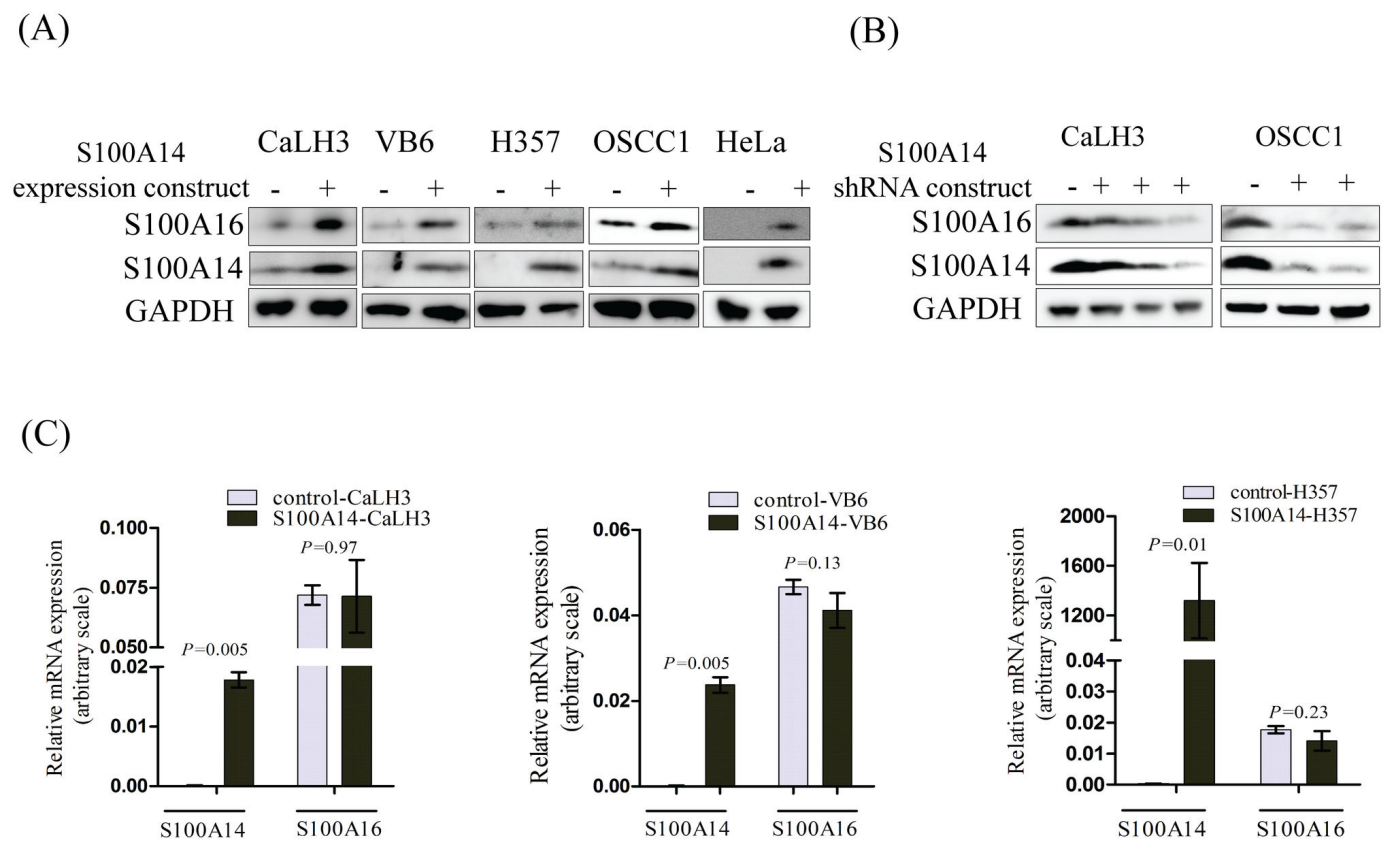
Over-expression of S100A14 modulates expression of S100A16 protein but not mRNA in human cancer cell-lines. (A) Retroviral mediated over-expression of S100A14 was associated with concomitant up-regulation of S100A16 protein in CaLH3, VB6, H357, OSCC1 and HeLa cell-lines (-, control construct; +, S100A14 expression construct). (B) Additionally, shRNA mediated knock-down of S100A14 in CaLH3 and OSCC1 cell-lines was associated with down-regulation of S100A16 protein (-, control construct; +, S100A14 shRNA constructs). (C) However, there was no effect in the mRNA expression levels of *S100A16* by over-expressing S100A14 in CaLH3, VB6, and H357 cell-lines. *S100A14* and *S100A16* mRNA expression levels were normalized to *GAPDH* mRNA expression. Error bars represent SEM of 3 biological replicates done in 3 technical replicates. Student’s-*t* test (paired) was performed for statistical analysis.

The biological significance of the interaction between S100A14 and S100A16 was next examined by over-expressing S100A14 in a number of human cancer cell-lines (CaLH3, VB6, H357, OSCC1 and HeLa) using a retroviral mediated gene expression strategy. S100A16 protein levels were found to be increased in all of the cell-lines examined following the over-expression of S100A14 (Figure 3A). In addition, shRNA mediated knock-down of S100A14 was associated with the suppression of S100A16 protein in CaLH3 and OSCC1 cell-lines (Figure 3B). However, there was no increase in the mRNA expression levels of S100A16 in these S100A14 over-expressing cancer cell-lines (Figure 3C).

### S100A16 does not regulate the expression of *S100A14* mRNA or protein in human cancer cell-lines

**Figure 4:**
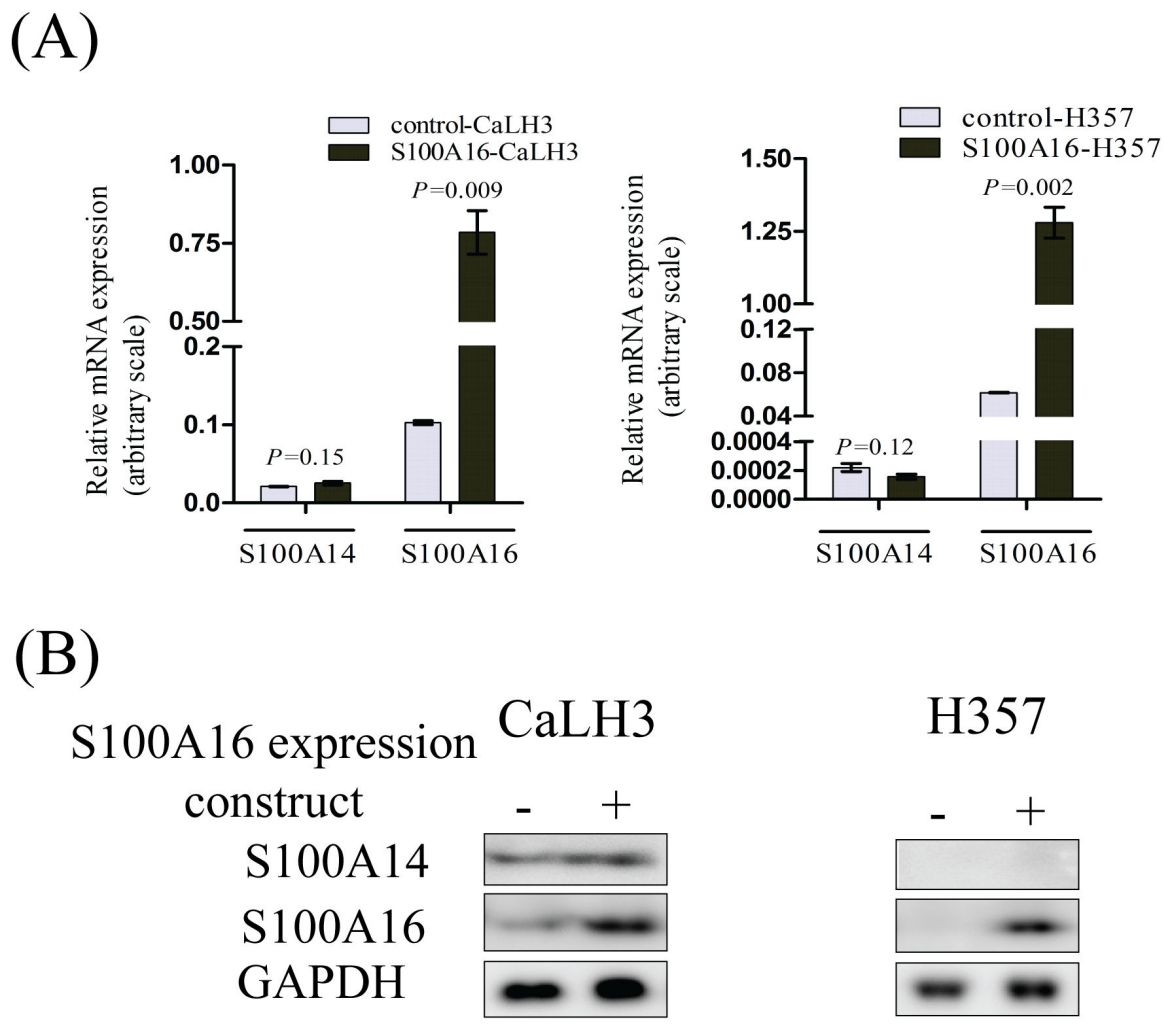
S100A16 regulates neither expression of *S100A14* mRNA nor S100A14 protein in human cancer cell-lines. There was no effect in the mRNA (A) or protein (B) expression levels of S100A14 by retroviral mediated over-expression of S100A16 in CaLH3 and H357 cell-lines (-, control construct; +, S100A16 expression construct). *S100A14* and *S100A16* mRNA expression levels were normalized to *GAPDH* mRNA expression. Error bars represent SEM of 3 biological replicates done in 3 technical replicates. Student’s-*t* test (paired) was performed for statistical analysis.

We next examined whether S100A16 over-expression is also associated with the concomitant change in the expression of S100A14 in cancer cell-lines. There was no appreciable change neither in the mRNA (Figure 4A) nor protein levels (Figure 4B) of S100A14 following the over-expression of S100A16 using retroviral expression constructs.

### Both S100A16 and S100A14 proteins are degraded intracellularly by mechanism(s) independent of proteasomal and lysosomal pathways

**Figure 5:**
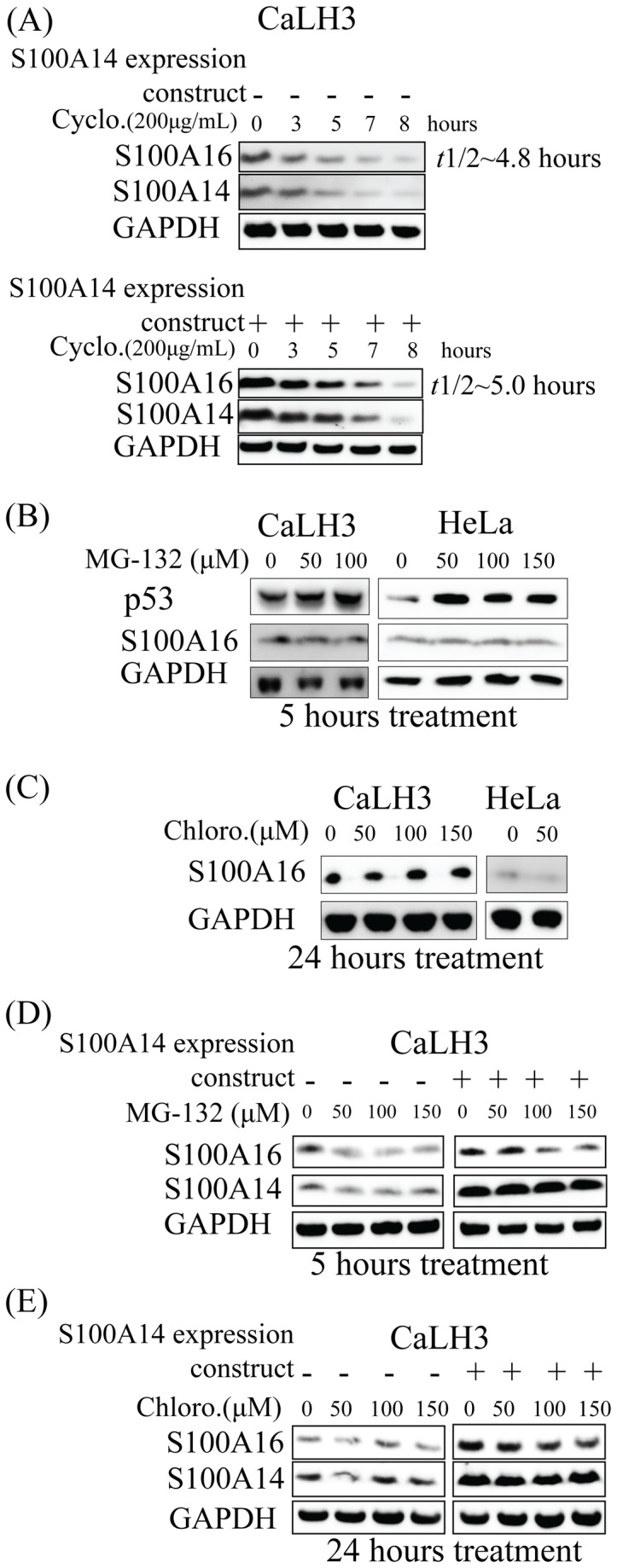
Both S100A16 and S100A14 proteins are degraded intracellularly by mechanism(s) other than proteasomal and lysosomal pathways. (A) Control and S100A14 over-expressing CaLH3 cells were treated with protein synthesis inhibitor cycloheximide in a time-course (0, 3, 5, 7 and 8 hours) and the whole cell lysates were immunoblotted with anti-S100A16 and -S100A14 antibodies. Relative S100A16 expression (normalized to GAPDH protein expression) was plotted against time-course and half-life (*t*1/2) was calculated for both control and S100A14 over-expressing CaLH3 cells (data not shown). -, control construct; +, S100A16 expression construct. Cyclo, cycloheximide. (B) CaLH3 and HeLa cell-lines were treated with various concentrations of proteasomal inhibitor MG-132 (0-150 µM) for 5 and 10 hours (data for 10 hours not shown) and whole cell lysates were immunoblotted with anti-S100A16 and -p53 antibodies. Treatment with MG-132 is associated with up-regulation of p53 (used as a positive control for the proteasomal inhibition experiments) but not S100A16 in both the cell-lines. (C) CaLH3 and HeLa cell-lines were treated with various concentrations of lysosomal inhibitor chloroquin (0-150 µM) for 24 hours and whole cell lysates were immunoblotted with anti-S100A16 antibody. Chloroquin treatment is not associated with change in the S100A16 expression in both the cells-lines. Concentrations higher than 50 µM of chloroquin resulted in excessive HeLa cell death and hence not used in the experiment. Chloro, chloroquin. (D) Control and S100A14 over-expressing CaLH3 cells were treated with various concentrations of proteasomal inhibitor MG-132 (0-150 µM) for 5 hours and whole cell lysates were immunoblotted with anti-S100A16 and -S100A14 antibodies. (E) Control and S100A14 over-expressing CaLH3 cells were treated with various concentrations of lysosomal inhibitor chloroquin (0-150 µM) for 24 hours and whole cell lysates were immunoblotted with anti-S100A16 and -S100A14 antibodies. Chloro, chloroquin.

Using cycloheximide treatment, time-dependent degradation of S100A16 and S100A14 was observed both in the control and S100A14 over-expressing CaLH3 cells (Figure 5A). There was not a significant difference in the S100A16 half-life (*t*1/2) between the control and the S100A14 over-expressing CaLH3 cells (~4.8 hours versus ~5.0 hours) (Figure 5A). Proteasomal and lysosomal pathways are involved in the degradation of the majority of intracellular proteins. The proteasomal inhibitor MG-132 effectively blocks the proteolytic activity of proteasome [22] and the lysosomal inhibitor chloroquin inhibits lysosomal hydrolases by reducing the acidification of endosomal/lysosomal compartments [23]. By treating the CaLH3 and HeLa cells with proteasomal (MG-132) and lysosomal (Chloroquin) inhibitors, we next examined whether these pathways were involved in the degradation of S100A16. No appreciable increase in the levels of S100A16 was observed after the inhibition of proteasomal (data for 10 hours treatment with MG-132 not shown) (Figure 5B) or lysosomal pathways (Figure 5C). The level of p53, used as a positive control for the proteasomal inhibition experiments, was increased as expected with the MG-132 treatment (Figure 5B). Additionally, no appreciable up-regulation of S100A16 and S100A14 was observed either in the control or in the S100A14 over-expressing CaLH3 cells with MG-132 (Figure 5D) or chloroquin treatments (Figure 5E).

## Discussion

Despite the diverse functional roles played by the S100 proteins, these proteins do not possess any enzymatic activity to account for their different cellular functions [3,4]. One of the mechanisms by which S100 proteins carry out their varied cellular functions is through interacting with and modulating the functions of other effector proteins. Recently, we [12,13] and others [14,15] have shown a functional role of S100A14 in human carcinogenesis. Involvement of S100A14 in the regulation of oral cancer cell proliferation and invasion, by modulating expression of several molecules including p21, MMP1 and MMP9, was reported by our recent studies [12,13]. These findings led us to investigate potential interaction partners of S100A14 which could modulate its cellular functions. Using Y-2H screen, we identified S100A16, another member of the S100 protein family, as the only true interaction partner of S100A14. This finding was rather surprising for several reasons. Firstly, considering its multiple functions in tumorigenesis with concomitant modulation of expression of several other molecules, S100A14 was expected to interact with many cellular proteins to execute its diverse functions. Secondly, the presence of N-myristoylation site at the N-terminus of the S100A14 protein suggests that this protein can interact with other proteins potentially involved in signal transduction [9]. Thirdly, S100A14 has already been shown to interact with other cellular proteins such as nucleobindin [10] and RAGE [14], but these proteins were not detected in the current Y-2H screen. However, findings of our current Y-2H screen are consistent with previous Y-2H screens for different S100 proteins. Similar to our findings, interacting partners for other S100 proteins from previous Y-2H screens are also largely dominated by the S100 protein isoform(s) (reviewed in 24). One of the suggested reasons for S100 isoform dominated interaction in Y-2H could be a function of tightly controlled intracellular Ca^2+^ concentration (200nM) in yeast cell [25] which is far below the calcium *K*_d_ values of many S100 proteins (10-50 µM) and that could limit interaction between S100 prey and its non-S100 interaction partners that require strict Ca^2+^ concentration for the interaction to take place. Nevertheless, in contrast to many other S100 proteins, structural analyses have suggested that both S100A14 and S100A16 are low affinity Ca^2+^ ion binders with minimal conformational changes after binding with Ca^2+^ [26,27] and hence strict Ca^2+^ concentration might not be necessary for the interaction between these two proteins. In fact, by increasing the Ca^2+^ concentration, increased immunoprecipitation was not observed between S100A14 and S100A16 in the current study (Figure 1A). However, no interaction was detected between S100A14 and S100A16 when 5mM of divalent metal ion chelator EDTA was used (Figure 1A). Taken together, these findings suggest that interaction between S100A14 and S100A16 is dependent on the basal levels of Ca^2+^ and possibly other divalent metal ions in the cells and increasing the concentration of Ca^2+^ does not necessarily enhance the interaction possibly because both S100A14 and S100A16 are poor Ca^2+^ binders with semi-open conformation even in apo-state and this conformation might not necessarily change even after binding Ca^2+^ ions.

Consistent with the findings of Y-2H and co-immunoprecipitation, co-localized expression of S100A14 and S100A16 was observed in the OSCCs and corresponding normal mucosa (Figure 2), indicating a possible functional significance of this interaction. Indeed, over-expression and knock-down of S100A14 in several cancer cell-lines was associated with concomitant up- and down-regulation of S100A16 protein (Figure 3A and B) but not mRNA (Figure 3C). These findings indicate that S100A14 regulates expression of S100A16 not at the transcriptional level but possibly by post-transcriptional or translational regulation. In contrary, no appreciable effect was observed both on the mRNA and the protein levels of S100A14 when S100A16 was over-expressed in cancer cell-lines (Figure 4A and B), suggesting a unidirectional regulation between S100A14 and S100A16 where only S100A14 regulates protein abundance of S100A16. Similar to S100A14, recent studies have shown differential expression of S100A16 in different human malignancies, suggesting a role of this protein in human cancers [28]. Considering the role of S100A14 in oral cancer cell proliferation and invasion [12,13] and its ability to regulate the expression of S100A16, it can be speculated that the S100A14\S100A16 heterodimer might have a functional significance in human cancer cells.

Having observed an increase only in the S100A16 protein and not in *S100A16* mRNA expression when S100A14 was over-expressed in the cancer cells, we speculated that S100A14 might be involved in the regulation of S100A16 protein degradation. The majority of eukaryotic cellular proteins (~80%) are degraded by two main proteolytic pathways- the proteasomal and the lysosomal degradation pathways [29–31]. However, some proteins are also degraded by other proteolytic mechanisms involving calpains [32] and caspases [33]. Following a time-course cycloheximide treatment, no significant difference in S100A16 half-life (*t*1/2) was observed between control and S100A14 over-expressing CaLH3 cells (Figure 5A). This observation initially suggested that the regulation of S100A16 expression might not be related to enhanced stability of S100A16 by S100A14 in S100A14 over-expressing cells. However, the concomitant degradation of S100A14 observed with cycloheximide treatment (Figure 5A) does not rule out the possibility that the interaction of S100A14 with S100A16 might stabilize or protect S100A16 from degradation in some way. Additionally, no appreciable increase in the levels of S100A16 and S100A14 was observed after the inhibition of proteasomal (Figure 5B and D) or lysosomal (Figure 5C and E) pathways. This indicates that intracellular degradation of S100A16 and S100A14 proteins is independent of the classical proteasomal and lysosomal pathways, at least, in the cell-lines examined. Hence, it will be a future challenge to examine how S100A14 is involved in the regulation of S100A16 expression and to understand the functional significance of S100A14/A16 heterodimer in normal and the cancer cells.
